# Antimicrobial resistance in *Escherichia coli* isolated from on-farm and conventional hatching broiler farms in Ireland

**DOI:** 10.1186/s13620-022-00214-9

**Published:** 2022-04-22

**Authors:** Noelle Byrne, Lorcan O’Neill, Julia Adriana Calderόn Dίaz, Edgar Garcίa Manzanilla, Ana P. Vale, Finola C. Leonard

**Affiliations:** 1 UCD School of Veterinary Medicine, College of Health and Agricultural Sciences, Dublin, Ireland; 2 Pig Development Department, Teagasc, Animal and Grassland Research and Innovation Centre, Moorepark, Fermoy, Co Cork Ireland

**Keywords:** Antimicrobial resistance, β-lactamases, Broiler, Hatching system, *E. coli*, Poultry

## Abstract

**Background:**

On-farm hatching (OH) systems are becoming more common in broiler production. Hatching conditions differ from conventional farms as OH chicks avoid exposure to handling, transport, post-hatch water and feed deprivation. In contrast, chicks in conventional hatching conditions (CH) are exposed to standard hatchery procedures and transported post hatching. The objectives of this pilot study were to investigate the prevalence and frequency of *Escherichia coli* resistant to antimicrobials, including presumptive ESBL/AmpC-producing *E. coli*, isolated from environmental and faecal samples from OH versus CH hatching systems, and to investigate the presence of ESBL/AmpC-producing encoding genes.

**Results:**

Environmental samples were collected from one flock in 10 poultry farms (5 OH farms, 5 CH farms) on day 0 post disinfection of the facilities to assess hygiene standards. On D10 and D21 post egg/chick arrival onto the farm, samples of faeces, boot swabs and water drinker lines were collected.

*E. coli* were isolated on MacConkey agar (MC) and MacConkey supplemented with cefotaxime (MC+). Few *E. coli* were detected on D0. However, on D10 and D21 *E. coli* isolates were recovered from faeces and boot swabs. Water samples had minimal contamination. In this study, 100% of cefotaxime resistant *E. coli* isolates (*n*=33) detected on selective media and 44% of *E. coli* isolates (84/192) detected on nonselective media were multidrug resistant (MDR). The antimicrobial resistance (AMR) genotype for the 15 ESBL/AmpC producing isolates was determined using multiplex PCR. Six of these were selected for Sanger sequencing of which two were positive for *bla*_CMY-2_, two for *bla*_TEM-1_ and two were positive for both genes.

**Conclusions:**

There was no difference in *E. coli* isolation rates or prevalence of AMR found between the OH versus CH systems, suggesting that the OH system may not be an additional risk of resistant *E. coli* dissemination to broilers compared to the CH systems. The frequency of β-lactam resistant *E. coli* in boot swab and faeces samples across both OH (24/33 (73%)) and CH (9/33 (27%)) systems may indicate that hatcheries could be a reservoir and major contributor to the transmission of AMR bacteria to flocks after entry to the rearing farms.

## Introduction

Antimicrobial resistance (AMR) is now recognised as one of the world’s most important health challenges [37]. Antimicrobial use (AMU) in agriculture and human and veterinary medicine is associated with an increase in AMR globally [33] and the misuse of antimicrobials in agriculture contributes to AMR to antimicrobials that are frequently used in human medicine [36]. The introduction of extended-spectrum cephalosporins (ESC) to human and veterinary medicine has improved the treatment of infection. However, resistance caused by extended spectrum β-lactamases (ESBL) and AmpC β-lactamase produced by *E. coli* and associated resistance to extended spectrum cephalosporins in poultry meat is a concern for public health [30].

Many studies have shown that the gastrointestinal tract of healthy broiler chickens can be a reservoir for antimicrobial resistant bacteria, particularly ESBL/AmpC-producing-*E. coli* [17, 32], with faeces, water, and litter acting as potential transmission sources [21]. Breeder flocks may be the original source of contamination, as one day old chicks are known to be a major risk factor for introduction of ESBL/AmpC-producing *E. coli* [11, 28] However, various important sources within the broiler production chain can contribute to the transfer of resistant bacteria to the birds on farm [20]. Transfer may begin at broiler grandparent/parent level, it may be from hatcheries or from residual contamination from the broiler rearing farms themselves [20, 23, 28]. Many factors are likely to influence the prevalence of resistant organisms within broiler production, such as: AMU, climate control, flock origin, hatchery, hygiene, nutrition, disease outbreaks/control, removal of waste (litter, devices, dead birds etc.), pest control and disinfection [15].

In the conventional hatching system (CH), chicks are hatched at the hatchery, vaccinated, and transported to the rearing farms before they can access any essential water or food; this period may last up to 48 hours. In the on-farm hatchery (OH) system, in contrast, eggs are transported to the rearing farm thus allowing the chicks immediate access to water and food directly after hatching. Aside from this, chicks that are born in hatcheries are exposed to environmental challenges and stressors such as dust, disinfection, and pathogen loads [9], as well as high noise level and continuous darkness [3], all of which are either reduced greatly or eliminated in an OH system. Such early life stressors can have long term consequences on the development and survival of chicks in later life [10]. In The Netherlands, a study reported improved health, welfare, and performance of chicks in the first week of life when reared in an OH system [9]. Another study investigated physiological differences in broiler chicks from hatching to day 45 in two different hatching systems: the ‘Patio’ system (hatching and brooding are combined) vs CH and concluded that either system had minor effects on hatching physiology [34]. Neither of these studies investigated AMR.

To date, published data relating to AMR associated with OH flocks in broiler houses and CH flocks in a hatchery are not available and therefore, the objective of this pilot study was to investigate the prevalence of antimicrobial resistant *E. coli* and AMR genes encoding clinically important β-lactamases, specifically ESBL/AmpC, in OH and CH broiler chickens and their rearing environment.

## Materials and methods

### Study Design

The study took place between October and December 2019. Ten broiler farms contracted by the same company, located in the same geographical region in the Republic of Ireland and served by the same hatchery participated in the study. The study batch on each farm consisted of one production round whereby five farms operated the conventional hatching system (CH farms) and five farms operated the on-farm hatching system (OH farms).

In the OH farms, pre-incubated eggs were transported from the hatchery to the broiler farms in setter trays and placed over a two-meter-wide strip directly onto a clean litter bed in the broiler rearing house by a self-propelled egg placing machine (Nestborn, Tienen, Belgium) approximately 3 days pre-hatching. The machine had two manual operators and a capacity to place 60,000 eggs per hour. The OH system completely removes the need for a hatchery service and eliminates the transport of live chicks. The CH farms used a standard method, whereby live chicks were transported directly to the rearing house on the farm after hatching in a hatchery. During the rearing period involved in this study, the birds did not receive antimicrobial treatment on any of the participating farms; vaccinations were administered as per the veterinary plan of the poultry company.

#### Sampling

Before chicks or eggs arrived at the farm (D0) environmental samples were taken from the rearing house to study baseline AMR in the *E. coli* isolates. On each farm, five individual samples from the floor, wall, vent, feeders, and drinkers were collected using sponge swabs (3M Health Care, Minneapolis, USA). The floor sample was collected by swabbing three areas of 1 m^2^ at the front, middle and end of the rearing house. Similarly, an area of 1 m^2^ was swabbed on the wall. One vent on the inside wall of the house was also swabbed at the front, middle and end of the rearing house as well as 5 clean feeders along a feeder line and 5 clean drinkers along a drinker line. Additionally, one clean litter sample, a feed sample (10 g) from one silo, pooled water sample (600 ml) from the water source and pooled water sample (600 ml) from the drinker end line were also collected. On OH farms, 5 swabs from external and internal areas of the egg placing machine were collected. Samples were stored in sterile containers and transported to the laboratory in cool boxes and processed on the same day of collection. Feed and clean litter samples were pooled by hatching method (i.e., OH and CH) for testing, because few *E. coli* were isolated from these samples in a pre-trial test run.

On day 10 (D10) and day 21 (D21) post egg/chick arrival, 20 g of freshly dropped faeces were taken from five distinct areas in each rearing house. D10 and D21 are the two main stages of feed change in the broiler rearing period and therefore were selected as sampling days. One pair of boot swab samples per house was also collected; a researcher walked from one end of the house and back again wearing pre sterilised boot swabs. The swabs were then stored back in the original packaging for microbiological examination. On D10 and D21, water sampling was repeated as per D0 procedure.

### Microbiological Analysis

#### Bacterial Isolation

Samples were stored at 4 °C during transport and on arrival at the laboratory. The sponge swab (5 pooled sponges), boot swab (one pair), faeces (20 g) (pooled/farm) and feed (10 g) samples were mixed with 300 ml, 180 ml, 180 ml, and 90 ml respectively of buffer peptone water (BPW) to make a 1:10 suspension and were homogenised using a stomacher (stomacher 400 circulator; Seward Limited, West Sussex, UK) for two minutes at 200 rpm. The homogenate from each sample was streaked onto one supplemented MacConkey agar (MC) plate and spread plated onto one supplemented (i.e., supplemented with 1mg/l cefotaxime MacConkey agar (MC+)) plate [2].

On day 0, water samples were collected in 500ml sterile containers from the source and drinker lines on each farm. The samples were transported from the farm in a cooler box and refrigerated at 4 °C on arrival to the lab on the same day. The desired volume of water was measured and then filtered aseptically using a vacuum pump through sterile, cellulose nitrate membrane filters, 47mm diameter and pore size 0.45μm. A total of 100 ml of each water sample was filtered through three separate sterile filters. Using a sterile forceps, a single filter with the grid side up was placed on two non-supplemented and one supplemented MacConkey agar plate. A newly sterilised water funnel was used to filter each water sample from each farm. A filter sterility check was performed by placing one membrane filter on a MacConkey plate and incubating it at 35°C ± 0.5°C for 24 ± 2 hours; absence of growth indicated sterility of the batch of filters.

All samples on MacConkey agar plates were incubated at 37 °C for 22 hours. Up to five suspect colonies with typical *E. coli* morphology from each of the MC and MC+ agars were transferred onto Tryptone Bile X-glucuronide (TBX) agar and TBX supplemented with cefotaxime (TBX+) and incubated at 37°C for 22 hours; *E. coli* isolates appear as blue colonies on TBX. Isolates that did not present typical *E. coli* morphology on TBX were further tested for production of indole and failure to utilise citrate. Selected isolates were sub-cultured on sheep blood agar to ensure purity before they were preserved onto cryo-beads (TSC Technical Service Consultants, UK) at -70 °C for future antimicrobial susceptibility testing (AST).

### Antimicrobial Susceptibility Testing (AST)

The BioMérieux VITEK2 system was used to test antimicrobial susceptibility of the isolates using the AST GN98 card. Isolates were resuscitated onto sheep blood agar from frozen and incubated at 37 °C for 24 hours. A panel of 17 antibiotics was tested: ampicillin (AMP), amoxicillin/clavulanic acid (AMC), cefalexin (CEF), cefpodoxime (CPD), cefovecin (CEV), ceftazidime (CTZ), ceftiofur (CFR), imipenem (IMP), amikacin (AMK), gentamicin (GEN), ciprofloxacin (CIP), enrofloxacin (ENR), marbofloxacin (MAR), doxycycline (DOX), nitrofurantoin (NIT), chloramphenicol (CHL) and trimethoprim/sulfamethoxazole (SXT). *Escherichia coli* ATCC 25922 was used for quality control.

Results were interpreted according to the criteria used by the VITEK software as shown in Table 1. Vitek criteria are based on those of CLSI [5, 6].

**Table 1:** Summary of antimicrobials included in the Vitek 2™ AST-GN 98 card and respective breakpoints used

Antimicrobial	Range (mg/L)	Breakpoints (mg/L)		
		S	I	R
Amikacin	2 - 64	<=4	8	>=16
Amoxicillin/Clavulanic Acid	2 - 32	<=8		>=16
Ampicillin	2. - 32	<=8		>=16
Cefalexin	4 – 64	<=4		>=8
Ceftiofur	1 – 8	<= 2		>=8
Cefovecin	0.5 - 8	<= 2	4	>=8
Cephalothin	2 - 64	<= 2	4	>=8
Chloramphenicol	2 - 64	<=8	16	>=32
Doxycycline	0.5 - 16	<=4	8	>=16
Enrofloxacin	0.12 – 4	<=0.5	1-2	>=4
Gentamicin	1 – 16	<= 2	4	>=8
Marbofloxacin	0.5 - 4	<=1	2	>= 4
Nitrofurantonin	1 - 16	<=64		>=64
Tetracycline	1 - 16	<=4	8	>=16
Trimethoprim/Sulphamethoxazole	20 – 320	<=40		>=80

VITEK2 software reports presumptive ESBL. Isolates displaying resistance to three or more antimicrobial classes were considered MDR [22]. The resistance status of each isolate was recorded as a binary variable, either susceptible (S) or resistant (R). Isolates with an intermediate (I) status were considered resistant (R).

### Investigation of β-Lactamase Encoding Genes using Multiplex PCR Technique

Presumptive ESBL/AmpC-producing *E. coli* were tested for β-lactamase-encoding genes using the multiplex PCR methods described by [7]. Rapid DNA extraction was performed on these isolates by the boiling method [7]. Each isolate was tested for: β-lactamase encoding genes (TEM (800bp), OXA (564bp), and SHV (713bp)) (Multiplex I); ESBL (CTX-M Groups 1 (688bp), 2 (404bp) and 9 (561bp), 8/25 (326bp) (Multiplex II); plasmid mediated AmpC (ACC (346bp), FOX (162bp), MOX (895bp), CIT (538bp), DHA (997bp) and EBC (683bp)) (Multiplex III); and VEB (648bp), PER (520bp), GES (399bp) (Multiplex IV). Positive control strains were obtained from Galway University Hospital National Microbiology Reference Library.

Each multiplex PCR reaction (I, II, III and IV) was carried out as per Dallenne et al. [7]. Briefly, PCR reactions [final volume: 25 μl (23.5μl master mix (MM) & template DNA (1.5 μl))] were set up with (2X QIAGEN multiplex MM, template DNA, RNase-free water, and primers). Primers were suspended to the appropriate concentration (Multiplex I: 0.4 pmol /10 μl, Multiplex II: 0.2 pmol /10 μl, Multiplex III: 0.2 pmol /20 μl and Multiplex IV: 0.3 pmol /10 μl,). For amplification, thermocycling was programmed in the following steps: template denaturation for 15 mins at 95 °C (1 cycle), 30 sec at 94 °C (30 cycles), annealing 90 sec at 60 °C (30 cycles), 90 sec at 72 °C (30 cycles) and final elongation 10 mins at 72 °C (1 cycle).

Gel electrophoresis was performed at 100 V for 2.5 hours, using a 2 % agarose gel and SYBR SAFE DNA gel stain. To visualise the DNA bands a UV transilluminator (Model: SYNGENE G: BOX) was used. Selected PCR products successfully amplified were sequenced using the Sanger sequencing method to determine the targeted β-Lactamase gene(s). Sanger sequencing is required to fully identify the specific β-lactamase gene targeted as the TEM primers target TEM variants including TEM-1 and TEM-2 (see [7], Multiplex I), and the CIT primers target LAT-1 to LAT-3, BIL-1, CMY-2 to CMY-7, CMY-12 to CMY-18 and CMY-21 to CMY-23, (see [7], Multiplex III). Sequence analysis was performed using Reverse Compliment (https://www.bioinformatics.org/sms/rev_comp.html) and nucleotide BLAST (https://blast.ncbi.nlm.nih.gov/Blast.cgi?PROGRAM=blastn). Comprehensive Antibiotic Resistance Database (CARD) (https://card.mcmaster.ca) was used to compare the nucleotide sequence queries with known β-Lactamase gene sequences by multiple sequence alignment.

### Statistical analysis

The percentage resistance of *E. coli* isolates to each antimicrobial was calculated in Excel. Differences between samples from OH and CH broiler operating system were analysed using the Mann Whitney U test using SAS 9.4 (The SAS Institute, Carey, NC). Alpha level for determination of significance was 0.05.

## Results

Total number of isolates recovered from non-selective (MC) and selective (MC+) media by hatching system, sample type and sample day (D) are shown in Table 2. No *E. coli* were recovered from the faecal samples collected from one OH farm on D21 when plated on MC or MC+ media despite the fact *E. coli* isolates were detected on the same farm in faeces sampled on D10 (Table 2).

**Table 2:** Total *E. coli* isolates detected on non-selective (MC) and selective (MC+) media in samples collected from low AMU poultry farms (*n*=10) in Ireland using either on-farm or conventional hatching systems

		MacConkey agar (MC, non-selective)				MacConkey agar with cefotaxime (MC+, selective for presumptive ESBL/AmpC+ ***E. coli***)			
		Hatching system				Hatching system			
Sample day	Sample type	Conventional hatching		On-farm hatching		Conventional hatching		On-farm hatching	
		No of isolates	No of farms (5)	No of isolates	No of farms (5)	No of isolates	No of farms (5)	No of isolates	No of farms (5)
**D0**	**Sponge Swab**	0^a^		1^a^	1	0		0	
	**Water (Source)**	1	1	0		0		0	
	**Water (Line End)**	0		0		0		0	
	**Feed**	0		0		0		0	
	**Clean Litter**	1	1	0		0		0	
**D10**	**Faeces**	25	5	25	5	0		10	2
	**Water (Line End)**	5	3	1	1	0		0	
	**Boot swab**	25	5	25	5	0		7	2
**D21**	**Faeces**	25	5	20	4	5	1	0	
	**Water (Line End)**	12	3	5	2	0		0	
	**Boot Swab**	25	5	22	5	4	1	5	2
**Total**		119		99		9		22	

^a^When present, up to 5 presumptive *E. coli* isolates per sample were identified and stored

On D0, few *E. coli* isolates were detected in environmental samples which included feed from the silo, clean litter, sponge swabs, water from the source and the drinker lines. All samples were negative on both MC and MC+ media for both hatching systems with the following exceptions from MC media: one CEF resistant isolate from a sponge swab sample from one OH farm, one CEF resistant isolate from a water (source) sample from one CH farm and one resistant isolate (AMP, AMC, CEF, DOX, SXT) from a clean litter sample from the same CH farm. On another OH farm, a single *E. coli* isolate with CEF, CIP, ENR, MAR, DOX, CHL resistance was detected by sponge swab sampling of the egg placing machine.

Presumptive ESBL/AmpC-producing *E. coli* were isolated on two farms. Presumptive ESBL/AmpC*-*producing *E. coli* isolates were detected in faecal and boot swab samples on MC+ media from one OH farm at D10 but were only detected in boot swab samples from the same farm at D21 (Table 2). *E. coli* were isolated on MC+ media from faecal and boot swab samples collected on one CH farm on D21 in contrast to D10 when it was not detected (Table 2). The same two farms of the five OH farms investigated were positive on MC+ media for presumptive ESBL/AmpC-producing *E. coli* isolates on both D10 and D21. Presumptive ESBL/AmpC *E. coli* producing isolates on MC+ media were recovered from boot swab (*n*=4) and faeces (*n*=5) on a single CH farm on D21 (Table 2).

**Fig. 1:**
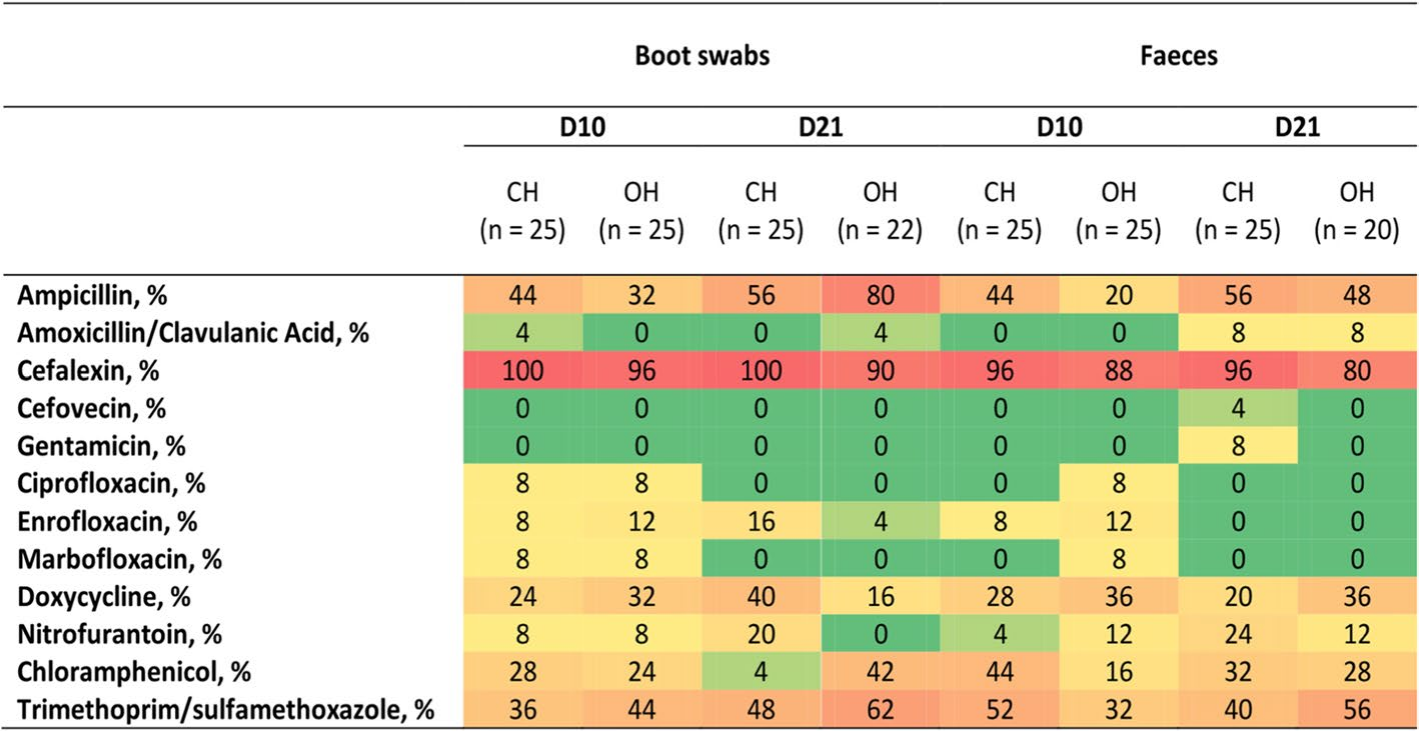
Heat map displaying the resistance percentage of *E. coli* isolates recovered on (MC) media according to sample type and rearing stage. Samples were collected from five farms with conventional hatching (CH) and five farms with on-farm hatching (OH), both with low antimicrobial use. ^a^(OH = on-farm hatching; CH = conventional hatching). Resistance to imipenem, amikacin, ceftazidime, cefpodoxime or ceftiofur was not detected in any isolate

The percentage of *E. coli* recovered on MC media from boot and faecal swabs collected on farms with OH and CH rearing systems, that were resistant to selected antimicrobials on D10 and D21 are presented in Fig. 1. Resistance to cefpodoxime, ceftazidime, ceftiofur, imipenem and amikacin was not detected in any isolate recovered from non-selective plates. There were no significant differences (Mann Whitney U test) between the two hatching systems in resistance to any of the antimicrobials tested. Resistance to cefalexin was high in *E. coli* isolated from all sample types and days, followed by resistance to ampicillin, trimethoprim, doxycycline and chloramphenicol.

**Fig. 2:**
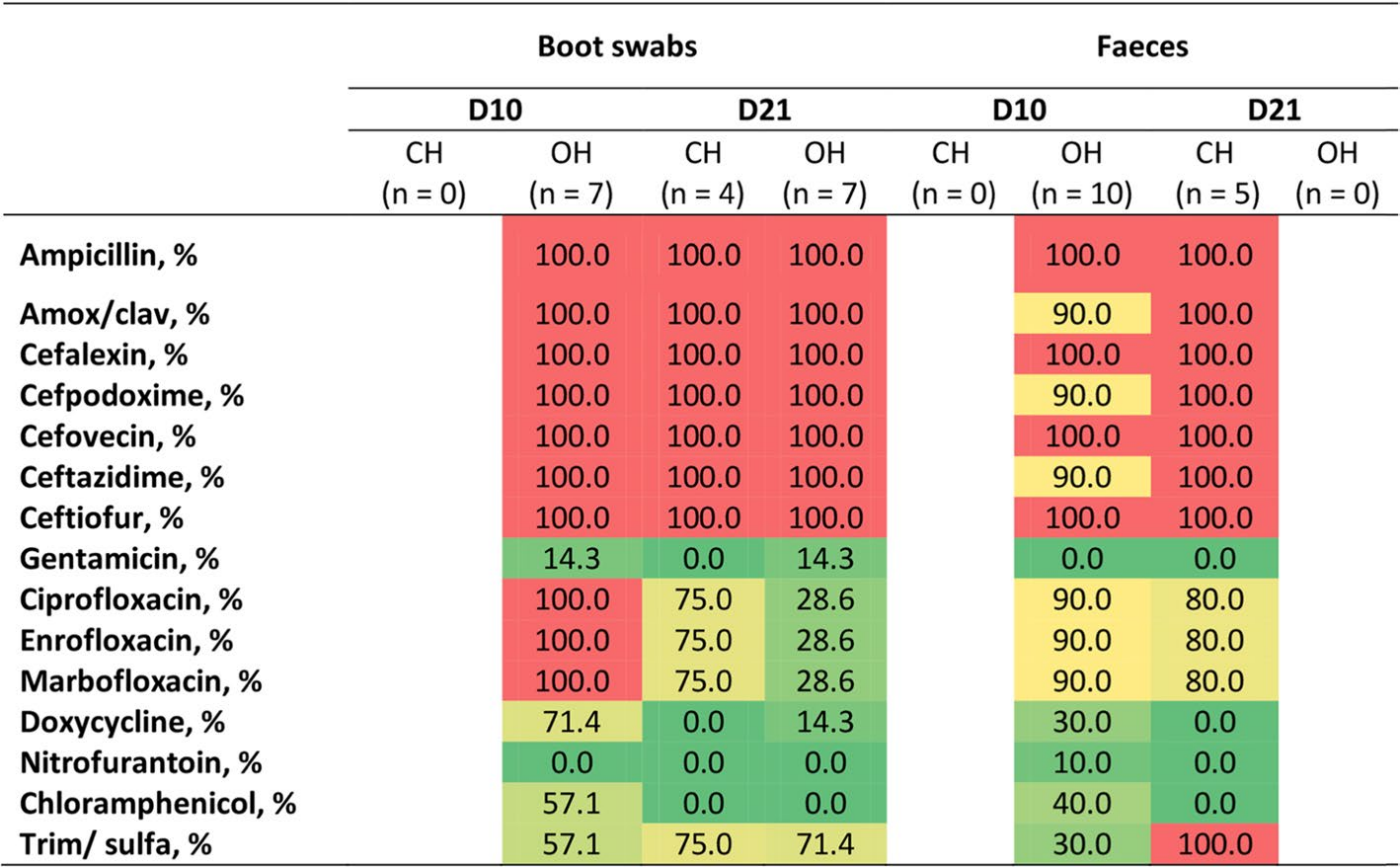
Heat map displaying resistance percentage of *E. coli* isolates recovered from faeces and boot swab samples on MC+ media from one farm with conventional hatching (CH) and two farms with on-farm hatching (OH) with low antimicrobial use. Resistance to amikacin or imipenem was not detected in any isolate. Amox/clav - amoxicillin/clavulanic acid; Trim/sulfa - trimethoprim/sulfamethoxazole

Resistance in *E. coli* recovered from selective media on D10 and D21 is presented in Fig. 2. Presumptive ESBL/AmpC-producing *E. coli* were detected on both D10 and D21 in samples from two OH farms and in addition they showed high levels of resistance to tetracycline, fluoroquinolones and potentiated sulphonamides. One CH farm had cefotaxime-resistant *E. coli* present at D21 in boot swab and faeces samples but did not have any cefotaxime-resistant *E. coli* present in samples collected on D10. The highest resistance percentage was seen in boot swab samples from OH farms at D10.

Resistance to imipenem and amikacin was not found on any farm at any sampling point. In this study, 84 out of 192 (44%) isolates recovered from MC media were MDR and all of the 33 ESBL/AmpC-producing isolates recovered from the MC+ plates were MDR.

The number and percentage of AMR *E. coli* isolates with specific resistance patterns that were detected on MC media on D10 and D21 from boot swab and faeces samples are shown in Table 3.

**Table 3:** Antimicrobial resistance patterns of 192 isolates recovered from boot swab and faeces samples on MC media on D10 and D21, post arrival of egg/chick onto on-farm hatching farms (n = 5) and conventional hatching farms (*n* = 5)

AMR PROFILE	Number of isolates	% Isolates
CEF	60	31%
AMP, CEF, CHL, SXT	24	12%
AMP, CEF, SXT	13	7%
AMP, CEF, DOX, SXT	11	6%
AMP, CEF	10	5%
CEF, ENR, DOX	7	4%
AMP, CEF, DOX, NIT, SXT	6	3%
AMP, CEF, DOX, CHL, SXT	6	3%
CEF, DOX, CHL, SXT	5	3%
CEF, DOX	5	3%
CEF, CHL	4	2%
CEF, NIT	4	2%
Other patterns found in 3 isolates or less	35	18%
Fully susceptible	2	1%
**Total**	192	100%
MDR	84	44%

*AMP* ampicillin, *AMC* amoxycillin clavulanic acid, *CEF* cefalexin, *CPD* cefpodoxime, *CEV* cefovecin, *CTZ* ceftazidime, *CFR* ceftiofur, *CHL* chloramphenicol, *CIP* ciprofloxacin, *ENR* enrofloxacin, *DOX* doxycycline, *GEN* gentamicin and *SXT* trimethoprim sulfamethoxazole

Numbers and percentage of AMR *E. coli* isolates with specific resistance patterns that were detected on selective MC+ media on sampling periods D10 and D21 from boot swab and faeces sample types are presented in Table 4. All the 33 isolates recovered were MDR.

**Table 4:** Antimicrobial resistance patterns of 33 isolates recovered from boot swab and faeces samples on MC+ media on D10 and D21, post arrival of egg/chick onto on-farm hatching farms (*n* = 2) and conventional hatching farm (*n* = 1)

AMR PROFILE	Number of isolates	% Isolates
AMP, AMC, CEF, CPD, CEV, CTZ, CFR, CIP	9	28%
AMP, AMC, CEF, CPD, CEV, CTZ, CFR, CHL, CIP, DOX, SXT	7	21%
AMP, AMC, CEF, CPD, CEV, CTZ, CFR, CIP, SXT	7	21%
AMP, AMC, CEF, CPD, CEV, CTZ, CFR, SXT	6	18%
AMP, AMC, CEF, CPD, CEV, CTZ, CFR, CIP, DOX, GEN	2	6%
AMP, AMC, CEF, CPD, CEV, CTZ, CFR	1	3%
AMP, CEF, CEV, CFR, CHL, NIT	1	3%
**Total**	33	100%

All isolates resistant to CIP were also resistant to ENR and MAR

*AMP* ampicillin, *AMC* amoxycillin clavulanic acid, *CEF* cefalexin, *CPD* cefpodoxime, *CEV* cefovecin, *CTZ* ceftazidime, *CFR* ceftiofur, *CHL* chloramphenicol, *NIT* Nitrofurantoin, *CIP* ciprofloxacin, *ENR* enrofloxacin, *NIT* nitrofurantoin, *DOX* doxycycline, *GEN* gentamicin and *SXT* trimethoprim sulfamethoxazole

### Investigation of β-lactamase-encoding genes

**Fig. 3:**
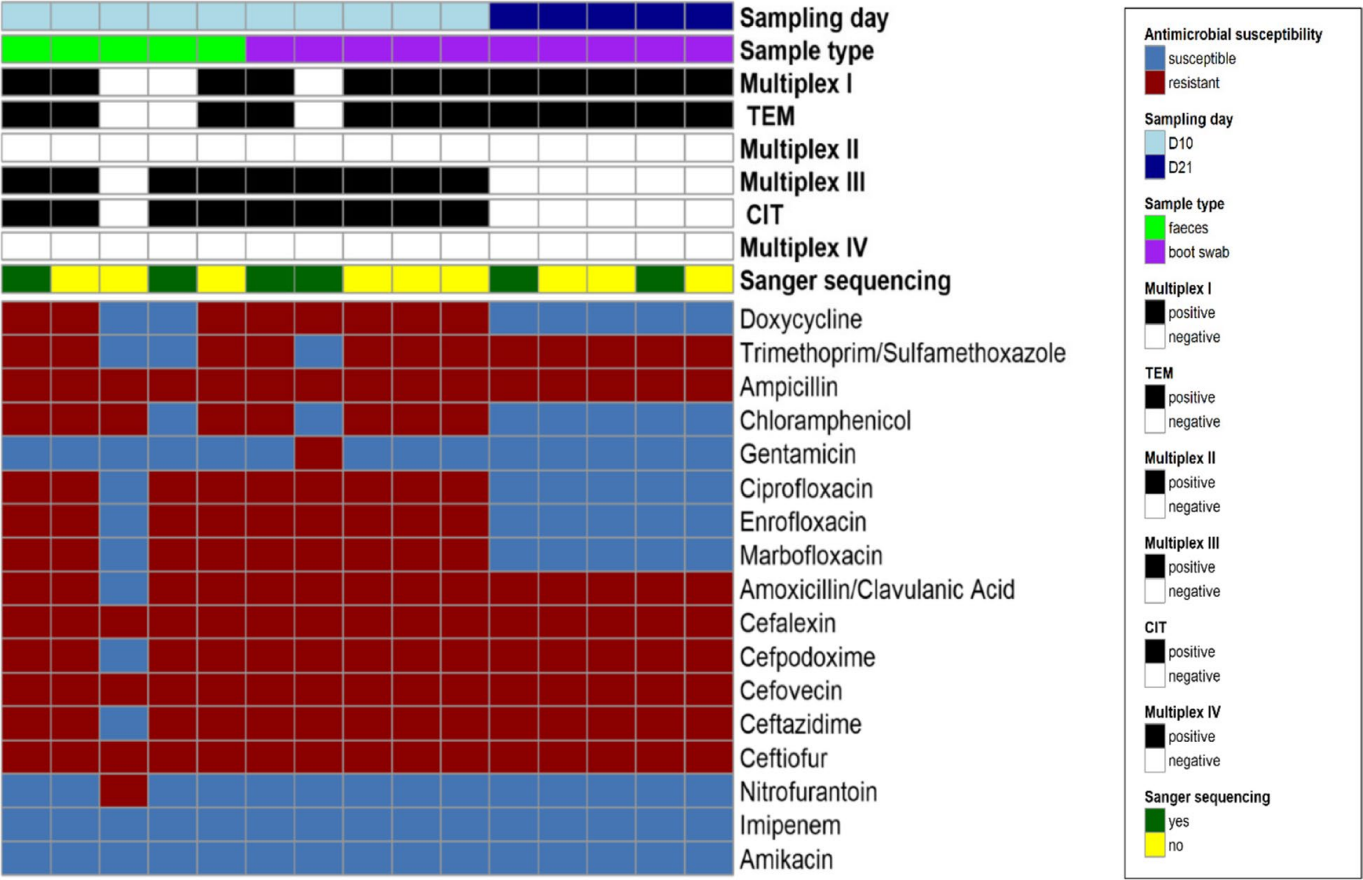
Heat map displaying antimicrobial resistance susceptibility patterns of presumptive ESBL/AmpC-producing *E. coli* recovered on selective media (MC+) according to sample day (D10 and D21) and type (boot swab and faeces). Selected presumptive ESBL/AmpC-producing *E. coli* isolates were tested for β-lactamase-encoding genes using the multiplex PCR method. Multiplex I targeted TEM, SHV and OXA-1-like β-lactamases, multiplex II targeted CTX-M group 1, 2, 9 and 8/25 β-lactamases, multiplex III targeted plasmid mediated AmpC β-lactamases and multiplex IV targeted VEB, PER and GES β-lactamases. Six of the *E. coli* isolates with a PCR product amplification were subsequently sent for Sanger sequencing to fully identify the specific β-lactamase gene targeted: *bla*_TEM-1_ was detected in all multiplex I positive samples; *bla*_CMY-2_ in all multiplex III positive samples. Each column represents the data of a single isolate

Fifteen presumptive isolates from the farm with the most positive samples were selected for testing using multiplex PCR. The PCR multiplex results and AST profile for all 15 isolates are illustrated in Fig. 3. On D10, seven out of 10 isolates were positive in multiplex I and all but one was positive in multiplex III. No isolate was positive in multiplex II or IV while one isolate was negative in all four multiplexes. In contrast, all five isolates from D21 were positive in multiplex I only. In the D10 samples, only the isolate negative in all multiplexes was susceptible to fluoroquinolone whereas all isolates from D21 were susceptible. Two of the isolates that were positive in each multiplex and two of the isolates that were positive in both multiplex I and multiplex III were submitted for Sanger sequencing (six in total). The gene detected in multiplex I was identified by sequencing as *bla*_*TEM-1*_ (GenBank Accession no: 30003984) for all four positive isolates and the gene detected in multiplex III was *bla*_*CMY-2*_ (GenBank Accession no: 3003138) for all four positive isolates*.*

## Discussion

The detection of antimicrobial resistant *E. coli* in the environment and the microbiome of broiler chickens is becoming more common and a cause for concern [31]. Such detection shines a light on the importance of routine investigation of commensal bacteria in broiler production in Ireland and elsewhere. The ‘One Health’ concept recognises that the health of humans, animals and the environment is interlinked with potential crossover of AMR reservoirs, especially *E. coli,* between different settings [25]. In Ireland, levels of antimicrobial resistant *E. coli* isolated from broilers are declining; this gradual trend may be reflective of restricted AMU practice in recent years. As per the [13] report, a decline in the occurrence of resistance to AMP, CIP and TET in *E. coli* isolated from broilers has been detected. Nevertheless, high levels of resistance to these antimicrobials were found in this study. In the most recent report based on 2019 data submitted from Ireland, levels of resistance of 35.9%, 5.3% and 57.1% to AMP, CIP and TET respectively were recorded in poultry, with MDR *E. coli* at 38.2% [13]. A total of 84/192 (44%) of *E. coli* isolates from MC plates in this study were MDR, which is higher than the EFSA figure. However, findings from this report [13] are based on data collected from broilers at slaughter in a nationwide study and using harmonised ECOFFs whereas, the findings in this study represent ten farms and use clinical breakpoints as set by the VITEK2. Therefore, direct comparison of findings is not possible.

Resistant strains of *E. coli* may persist in farm environments and rapidly colonise new flocks [11, 20]. Recent studies have described various modes of transmission of antimicrobial resistant *E. coli* from the hatchery environment and along the entire broiler rearing process [12, 29]. However, although some studies examining production and other data in chickens reared in OH or CH intensive broiler rearing systems have been published, none have compared antimicrobial resistance in *E. coli* recovered from these systems. Of the ten farms studied, *E. coli* was consistently detected on both CH an OH farms on D10 and D21. We hypothesised that levels of antimicrobial resistance in *E. coli* in the OH system would be lower than in the CH systems because of the elimination of hatching on other premises and avoidance of transportation, thus reducing possible exposure of chicks to sources of antimicrobial resistant organisms and stress. Under the conditions of this study, no differences were observed in recovery of resistant *E. coli* between OH and CH farms. Nevertheless, high levels of MDR *E. coli* isolates were recovered on D10 and D21 on both farm types. The isolation rates of *E. coli* on OH farms from this study may raise concerns about the introduction of antimicrobial resistant *E. coli* to chicks via the grandparent/parent lines or in the eggs, in the steps before the hatching period on the rearing farms. For example, a study in 2008 reported that the risk of introducing ESBL/AmpC-producing *E. coli* to the flock was associated with purchasing chicks that were already positive due to clonal spread in grandparents and parent flocks [23]. Persistence of AMR on farms and transmission and movement of antimicrobial resistant organisms may occur through national or international importation of breeding stock to hatcheries. Furthermore, the repeated introduction of antimicrobial resistant organisms to young chicks from persistence within the hatchery and movement between premises via stock, transport vehicles, personnel, or wind is also possible [8]. In addition, the detection of *E. coli,* including cefotaxime resistant isolates, on OH farms may suggest transmission of antimicrobial resistant *E. coli* from eggshell contamination in the hatchery before or after shell disinfection. This theory is supported by [28] who concluded that egg-shell surfaces can continue to serve as a source of contamination even after disinfection. However, Hiroi et al. [19] and Laube et al. [20] suggested that environmental contamination of houses and insufficient disinfection were the cause of high levels of antimicrobial resistant *E. coli* in broiler faeces*.* In the study reported here, on D0, only four isolates of *E. coli* were recovered in environmental samples on two of the ten farms tested, suggesting that on-farm cleaning and disinfection between batches was highly effective. However, on D0, one MDR *E. coli* isolate was detected on the egg placing machine while it was in use; this suggests contamination between houses or farms due to insufficient disinfection could occur as the machine was used on multiple farms each day.

On D10, *E. coli* isolates were recovered from water drinker lines on four farms and from five farms on D21, two of which were the same as on D10 (Table. 2). This finding suggests that a build-up of *E. coli* occurred in water lines over time, possibly associated with biofilm formation.

On many farms the contamination increased over time. Minimal evidence of faecal contamination was found at D0 (4 isolates) but a high proportion of MDR *E. coli* was found in samples collected on D10 and D21. Farms selected for participation in this study were already designated by the company for inclusion in OH or CH systems. All flocks were sampled concurrently and because all farms were contracted to the same company, management and operating systems were similar across all farms including feed to minimise confounding factors as much as possible during the study. Results obtained from this study show a high occurrence of antimicrobial resistant *E. coli* in broiler flocks from both rearing pathways.

Of the 33 ESBL/AmpC-producing *E. coli* isolates detected from MC+ media from boot swab and faeces samples all were MDR. On MC media 84 isolates (44%) were MDR, which highlights the presence of MDR *E. coli* in the microbiome and environment of the broilers. ESBL/AmpC-producing *E. coli* were not detected on the MC medium which shows that they were not abundant. The epidemiology of ESBL/AmpC-producing *E. coli* is complex and the result of this study enhances the knowledge of AMR in broiler flocks by evaluating the AMR patterns of *E. coli* and provides additional information to the data already available in different European countries [13]. Fluoroquinolone resistance was common in MDR isolates from broilers in this study as 18 of 33 (55%) of *E. coli* recovered on MC+ media were fluoroquinolone resistant. The frequent use of fluoroquinolones over many years may explain the high levels of resistance detected. In addition, high fluoroquinolone resistance detected in presumptive ESBL/AmpC isolates suggests co-resistance, with genes encoding fluoroquinolone resistance possibly being on the same mobile genetic element as the cefotaxime resistance encoding genes. An alternative explanation would be the presence of a chromosomal mutation in these isolates. The rapid spread of antimicrobial resistant *E. coli* that harbour mobile genetic elements encoding AMR, raises an ongoing and immediate issue due to the hypothesised transmission to humans by way of the food chain.

Results from this study were based on multiple isolates from pooled samples, this approach was considered acceptable to ensure an unbiased estimate of *E. coli* resistance prevalence. Cefotaxime is recommended for routine monitoring as it is proven to be an effective substrate for inclusion in media for detection of the common ESBLs including the CTX-M enzymes [12]. The study was limited as only a small number of farms were used to detect differences in OH and CH hatching systems, but data collected within this small study will be useful for future research.

There are frequent reports from countries across the EU presenting information on the dissemination of ESBL-producing *E. coli* in poultry [14]. β-lactamase encoding genes have been reported in *E. coli* isolated from Irish food producing animals [35, 38]. In this study, investigation into the presence of β-lactamase encoding genes and plasmid-encoded AmpC genes in cefotaxime resistant *E. coli* isolates was performed on one of the farms. On this farm, all but one of the isolates recovered on D10 were positive in multiplex III (*bla*_CMY-2_ positive in those that were submitted for Sanger sequencing), however, by D21 all isolates were negative in multiplex III. This suggests the two different cephalosporin resistant *E. coli* populations detected may be age dependant which could reflect different sources of transmission, for example, hatchery versus farm. Since this study was limited to just one flock on this farm and the genetic basis for cephalosporin resistance on the other farms was not determined, further investigation is required. The Sanger sequencing results suggest that positive multiplex I isolates carry the *bla*_TEM-1_ gene which does not explain cephalosporin resistance in the D21 isolates. The most likely explanation is chromosomal AmpC production which is a relatively common mechanism in some countries [26].

In poultry, CMY-2 is the most common plasmid-mediated AmpC β-lactamase in *E. coli* isolates of animal and human origin [18]. The prevalence of CMY-2-mediated cephalosporin resistant *E. coli* can differ depending on geographical region [16]. In *E. coli* isolates of animal origin, CMY-2 was the most frequently detected cephalosporin resistance determinant in Denmark (83%) and imported broiler meat (42%) until 2013 [1]. In 2014, a reduction in the occurrence of CMY-2 producing *E. coli* was detected in Danish (23%) and imported (33%) broiler meat, this change most likely comes from the discontinued use of 3^rd^ generation cephalosporins in hatcheries at the top of the broiler production pyramid [4]. The high prevalence of the *bla*_*CMY-2*_ gene in isolates from poultry is an important public health concern. Our results show, the *bla*_*CMY-2*_ positive isolates were MDR, including fluoroquinolone resistant. These findings are comparable to those of a study by [27]. Agersø [1] reported that, ESBL genes and ESBL producing *E. coli* clones carrying plasmids were detected in imported broiler parent flocks on a Danish conventional broiler farm, even though cephalosporins had never been used. Furthermore, in a Norwegian study, stable colonisation with *bla*_*CMY-2*_ producing *E. coli* was observed through an entire broiler production chain from grandparent birds to retail meat [24].

## Conclusion

Broiler chickens may be a reservoir for antimicrobial resistant *E. coli* from early life and thus, may facilitate transmission of antimicrobial resistant *E. coli* to other chickens in the same flock. Very few antimicrobial resistant *E. coli* isolates were detected on D0, but a greater number of samples were positive on D10 and D21 of the broiler rearing period, although antimicrobials were not administered to the birds at any time. Contrary to the initial theory on the benefits of the OH broiler rearing system, it did not appear to have reduced the prevalence of antimicrobial resistant bacteria in the flocks. As most of the antimicrobial resistant *E. coli* isolates were detected on D10 and D21 post arrival of eggs/chicks and not on D0, this may indicate transmission to the birds from the hatchery environment rather than from the rearing farms. Further investigation of this finding using a larger sample size would be useful.

The high prevalence of MDR *E. coli* is of concern in terms of resistance to critically important antibiotics and human health. Analysing phenotypic AMR of commensal *E. coli* from the environment and intestinal flora of broiler chickens can provide information on the reservoirs of AMR bacteria that may be disseminated between animal and human populations.

Results from this study suggest that important mitigation strategies should include more stringent biosecurity and disinfection measures in the hatchery and on farms, to prevent buying in broilers carrying resistance at the beginning of life and testing of imported breeding stock for antimicrobial resistant *E. coli* before entry to the breeding farms. Education of farmers and farm workers on the harmful impacts of AMR would also be of benefit.

## Data Availability

Not applicable.

## Abbreviations

AM: Antimicrobial
AMP: ampicillin
AM: amoxycillin clavulanic acid
AMR: Antimicrobial Resistance
AMU: Antimicrobial Use
AMURAP: Antimicrobial Use and Resistance in Animal Production
AST: Antimicrobial Susceptibility Testing
BPW: Buffer Peptone Water
BS: Boot Swab
CARD: Comprehensive Antibiotic Resistance Database
CEF: cefalexin
CEV: cefovecin
CFR: ceftiofur
CH: Conventional-Farm Hatching
CHL: chloramphenicol
CIP: ciprofloxacin
CPD: cefpodoxime
CLSI: Clinical & Laboratory Standards Institute
CTZ: ceftazidime
DAFM: Department of Agriculture, Food & the Marine
*E. coli*: *Escherichia coli*
EFSA: European Food Safety Authority
ESBL: Extended Spectrum Beta Lactamases
ESC: Extended Spectrum Cephalosporin
EUCAST: European Committee on Antimicrobial Susceptibility Testing
GN: Gram Negative
MIC: Minimum Inhibitory Concentration
MC: MacConkey Agar
MC+: MacConkey Agar plus cefotaxime 1 mg/L
MDR: Multi Drug Resistant
NCBI: The National Centre for Biotechnology
OH: On-Farm Hatching
SOP: Standard Operating Procedure
SXT: trimethoprim sulphonamide
TBX: Tryptone Bile X-glucuronide agar
